# Post-Stroke Recovery in Relation to Parvalbumin-Positive Interneurons and Perineuronal Nets

**DOI:** 10.1177/15459683241309567

**Published:** 2025-01-16

**Authors:** Lydia M. Kuhl, Matthew S. Jeffers, Nicolay Hristozov, Sudhir Karthikeyan, Matthew W. McDonald, Aisha Hufnagel, Anthony Carter, Numa Dancause, Dale Corbett

**Affiliations:** 1 Department of Clinical Neurosciences, Hotchkiss Brain Institute, University of Calgary, AB, Calgary, Canada; 2 Department of Cellular and Molecular Medicine, University of Ottawa, Ottawa, ON, Canada; 3 School of Epidemiology and Public Health, University of Ottawa, Ottawa, ON, Canada; 4 Clinical Epidemiology Program, Ottawa Hospital Research Institute, Ottawa, ON, Canada; 5 Département de Neurosciences, Université de Montréal, Montréal, QC, Canada; 6 Centre interdisciplaire de recherche sur le cerveau et l’apprentissage, Université de Montréal, Montréal, QC, Canada

**Keywords:** stroke, rats, recovery, motor behavior, critical window

## Abstract

**Background:**

There is a critical time window of post-stroke neuroplasticity when spontaneous behavioral recovery occurs. Potential factors responsible for this heightened plasticity are the reduction of parvalbumin-immunoreactive (PV+) interneuron inhibitory signaling and the disappearance of extracellular matrix synaptic stabilizers called perineuronal net(s; PNN/PNNs).

**Objective:**

This study investigated whether behavioral recovery during this critical period following stroke is associated with changes in densities of PV+ interneurons and PNNs.

**Methods:**

Male, Sprague-Dawley rats received forelimb motor cortex stroke (n = 43) using endothelin-1, or vehicle injections (n = 44). Cohorts of rats underwent a battery of motor tests and were sacrificed within the post-stroke critical window on day 1, and 1, 2, 4, and 6 weeks. Using immunofluorescent labeling, PNNs (wisteria floribunda agglutinin; WFA+ cells), PV+ interneurons, and cells expressing both PV and PNNs were quantified in contra- and ipsilesional cortices to elucidate their spatial-temporal profiles following stroke.

**Results:**

PV+ interneuron density decreased significantly at 1-day post-stroke in the lateral ipsilesional cortex, while the density of PNNs was significantly lower up to 4 weeks post-stroke in the lateral ipsilesional cortex and at 1 and 2 weeks post-stroke in the medial ipsilesional cortex. Reduction of combined PV+/PNN signaling coincided with spontaneous behavioral recovery.

**Conclusions:**

These results suggest that post-stroke behavioral recovery corresponds to an early reduction in PV+/PNN co-labeled cells in conjunction with an early temporally-dependent reduction in PV+ interneuron signaling and chronic disappearance of PNNs. Interventions targeting PNNs or PV+ interneuron signaling have significant potential for extending the critical window of recovery following stroke.

## Introduction

With advances in acute stroke care more patients are surviving stroke, albeit with persistent impairments, making stroke the second leading cause of disability worldwide.^1,2^ Thus, there is an urgent need for new therapies to further enhance recovery.^3-5^ In rodent stroke models, there is a critical window of increased neuroplasticity between 5 and 30 days post-stroke, coinciding with the time when rehabilitation is most effective.^6,7^ This window is characterized by an upregulation of growth factors (eg, BDNF and GAP43) associated with axonal sprouting and synaptogenesis^6,8,9^ and, subsequently, the reorganization of neuronal connections and networks.^9^ Perineuronal nets (PNNs), which are components of the extracellular matrix, are also known to be modified throughout this post-stroke critical window.^6,10^

PNNs play a role in critical periods of neurodevelopment,^11^ during which their emergence is associated with a reduction in neural plasticity.^12-15^ In addition, PNNs envelop many parvalbumin-immunoreactive (PV+) interneurons,^15-17^ where they help maintain spiking of these interneurons and inhibit neurite outgrowth.^18^ The PV+ interneuron subset of ϒ-aminobutyric acid (GABA) ergic interneurons facilitates inhibitory signaling^19-21^ and pharmacological attenuation of this signaling correlates with increased behavioral recovery after stroke.^22-24^ Following stroke, there is a decrease in the number and density of cortical PV+ interneurons^10,25,26^ and in the density of co-labeled PV+/PNN cells^10,27^ indicating less synaptic inhibition and presumably an opportunity for increased neuroplasticity.^10,14,27,28^

There is a lack of consensus on the timing of PNN and PV+ interneuron density fluctuations in the acute and subacute periods (ie, 3-30 days) post-stroke.^8,10,26,29^ Most studies that measure density of PV+ interneurons and PNNs use manual quantification methods which have been generally been limited to the ischemic core and perilesional regions of interest.^10,29^ Distal cortical areas that may be involved in recovery^25,30^ are often not included due to the time consuming nature of manual counting.^25^ Additionally, since quantifying cortical cells is a laborious process, previous studies^10,26,29^ have quantified PV+ interneurons and PNNs at a limited number of post-stroke timepoints.^10^

Following stroke, the critical window of neuroplasticity occurs in parallel with a period of spontaneous behavioral recovery.^6,31,32^ Since PNNs play a role in neuroplasticity and are influenced by stroke, it is important to document their temporal expression especially during the critical time window when most recovery occurs.^7^ To properly elucidate the temporal dynamics of PV+ interneurons and PNNs post-stroke, we quantified their densities throughout the cortex during and beyond the post-stroke critical window (>30 days) of neuroplasticity.^6,7^ The time course of PV+ interneuron and PNN density changes, in relation to behavioral recovery profiles, provides possible new insights into the contribution of PV+ interneurons and PNNs to the early determinants of post-stroke recovery.

## Methods

### Animals

**Figure 1.**
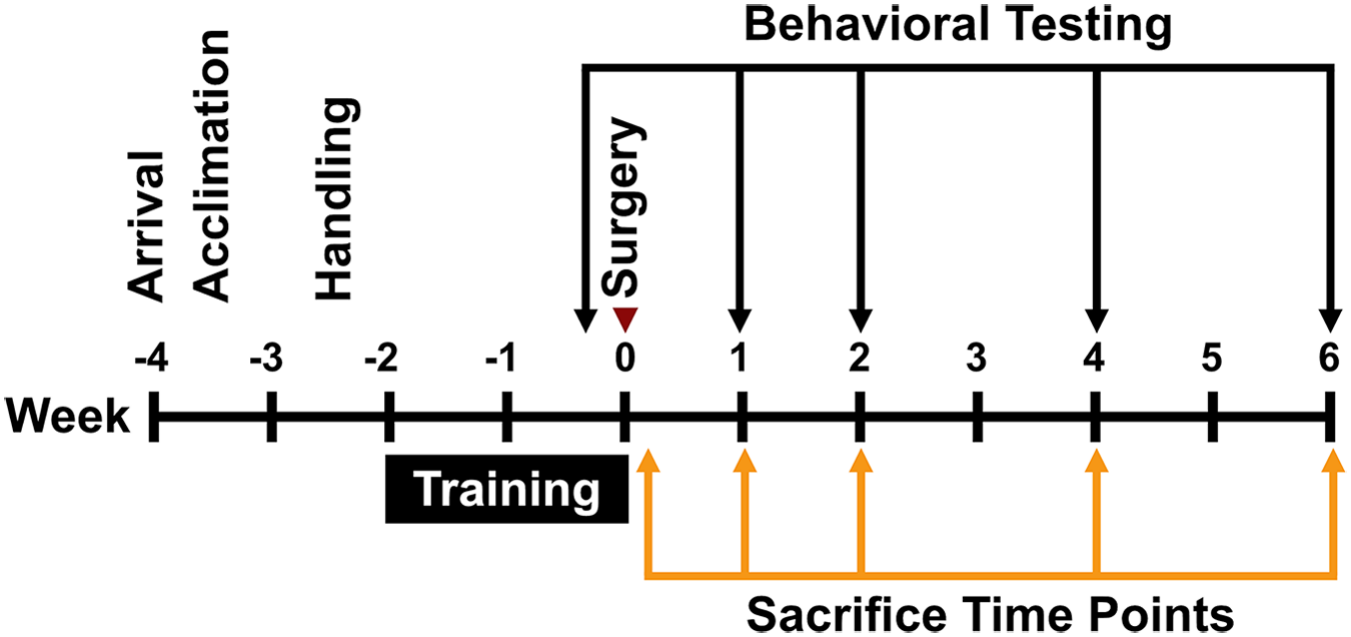
Experimental timeline.

A total of 91, ~3-month-old, male Sprague-Dawley rats (Charles River Laboratories, Montreal, Quebec, Canada) weighing 200 to 250 g at time of arrival were used in these experiments (for discussion of power, see Supplemental Methods: Section 1). Animals were acclimated to standard cage pair-housing for 7 days on a reverse 12-hour light/dark cycle, with ad libitum access to food and water (excluding behavioral training and testing periods). For the next 7 days the rats were handled daily (~5 minutes per day). The rats were then given 2 weeks of training/familiarization on the behavioral tests described below, after which they were randomly assigned and subjected to endothelin-1 (ET-1; n = 45) stroke or sham procedures (ie, ET-1 vehicle injection; n = 46). Two sham animals died from post-surgical complications, 1 stroke animal was excluded from the study due to bilateral damage and 1 animal was excluded because it lacked damage creating a final cohort of 87 rats. All experimental procedures (Figure 1) were approved by the University of Ottawa Animal Care Committee and complied with Canadian Council on Animal Care guidelines (protocol number CMM-2174-R1A1). Experimental procedures, statistical analyses, behavioral testing, documentation, and data reporting followed the ARRIVE 2.0 guidelines.^33^

### Behavior

After group assignment, all animals were trained/familiarized on 3 different behavioral tests: Montoya staircase, cylinder, and tapered beam. Post-surgery behavioral testing was done at 1, 2, 4, and 6 weeks for both stroke and sham groups. An experimenter blinded to surgery type carried out analyses of all behavioral tasks.

#### Montoya Staircase Test

The Montoya staircase test^34^ was used to assess forelimb reaching ability after stroke. Each pair of 7 steps contained 3 food pellets (45 mg; 5TUL, TestDiet) and, after each session (15 minutes duration), pellets were counted for each side of the staircase. The design of the staircase allowed animals to retrieve pellets on the left staircase using the left paw and vice versa. Pre-surgery training occurred twice per day for 14 consecutive days, with the criterion for inclusion being that each animal had to attain an average of 14 ± 2 pellets from the last 2 days of training. Post-surgery animals were given 1 day of practice to refamiliarize them with the task, and then tested for 2 consecutive days to assess impairments.

#### Cylinder Task

The cylinder test was used to assess asymmetry of ipsilateral and contralateral limb use.^35^ Animals were placed in a Plexiglas cylinder (20 cm diameter and 30 cm tall) on a glass surface. Video footage was captured from below, allowing the experimenter to assess upper limb contacts during rearing movements as the rats explored the cylinder. Each rat was filmed for 20 rears and, once testing concluded, the number of ipsilateral and contralateral forepaw touches to the cylinder wall were measured. Use of the contralateral limb was quantified as:

$$\%\;\;\mathrm{Use}\;\mathrm{of}\;\mathrm{impaired}\;\mathrm{paw}=\left(\frac{\begin{array}{l} \mathrm{Contralateral}\;\mathrm{contacts}\\ +\;\frac{1}{2}\;\mathrm{bilateral}\;\mathrm{contacts} \end{array}}{\mathrm{Total}\;\mathrm{contacts}}\right)\times 100\%$$

#### Tapered Beam Test

To assess forelimb and hindlimb placement impairments during locomotion, the tapered beam test was used.^36,37^ Rats were placed on a 160 cm long tapered beam (6.0 cm at wide point, 1.5 cm at narrow point), with a food reward box at the narrow end. During training, rats were placed at increasingly greater distances from the food reward until they traversed the beam consistently. Pre-stroke, animals typically traversed the beam with few foot faults (defined as a foot slipping off the beam), but foot-faults increased substantially post-stroke.^37^ The sum of foot faults across 4 trials was calculated at each test point. Comparing pre- and post-stroke foot faults allowed for an assessment of accuracy for impaired limb placement, and was calculated as follows:

$$\%\;\mathrm{Successful}\;\mathrm{steps}=\left(1-\frac{\mathrm{Foot}\;\mathrm{faults}}{\mathrm{Total}\;\mathrm{steps}}\right)\times 100\%$$

### Surgery

Following the end of the behavioral training period, and an overnight fast, rats were anaesthetized using isoflurane (4% induction, 2% maintenance in 100% oxygen) and positioned in a stereotaxic frame. Following a scalp incision, 2 burr holes were drilled in the skull (~1.0 mm diameter), and stereotaxic injections (relative to Bregma) of ET-1 or vehicle (sterile water) were made targeting the forelimb motor cortex (injection #1—AP = 0.0, ML = ±3.0, DV = −1.7; injection #2—AP = +2.3, ML = ±3.0, DV = −1.7) in the hemisphere opposite to the animal’s dominant forelimb as determined from the pre-stroke staircase testing. The stroke was induced with an injection of ET-1 (Abcam, ab120471) dissolved in sterile water at a concentration of 400 pmol/μL. A syringe (Hamilton, 80366) was lowered into the brain and, after 1 minute, 1 μL of ET-1 was injected at a rate of 250 nL/min followed by a 2-minute pause, prior to a slow withdrawal of the needle over a 2-minute period. Sham animals received the same procedure, but instead of ET-1 sterile water was injected.

### Histology

Sub-cohorts of rats were anaesthetized at 1 day, n = 17 (9 sham and 8 stroke); and 1 week, n = 18 (9 sham and 9 stroke); 2 weeks, n = 17 (9 sham and 8 stroke); 4 weeks, n = 18 (8 sham and 8 stroke); 6 weeks, n = 18 (9 sham and 10 stroke) post-stroke with euthanyl (Bimeda-MTC Health Inc.), 65 mg/kg (i.p.), followed by transcardial perfusion with heparinized saline, followed by cold 4% paraformaldehyde (PFA) in pH 7.4 phosphate-buffered saline (PBS). Heads were removed and stored in PFA for 24 hours to avoid dark neuron artifact.^38,39^ Brains were extracted and fixed for 24 hours in 4% PFA at 4°C before being transferred to 20% sucrose for 2-3 days for cryoprotection. The brains were stored at −20°C until sectioning at a thickness of 20 μm at an interval of 1:24 between sections on a cryostat. Mounted sections were dried overnight in a room-temperature chamber and stored at 4°C.

### Immunohistochemistry

Triple-label immunofluorescence staining was done for PV+ interneurons, PNNs, and neuronal cell bodies (for complete list of blocking solution components and antibodies used, see Supplemental Methods: Table 1). PNNs were visualized using Wisteria floribunda agglutinin (WFA), PV+ interneurons were visualized using an anti-parvalbumin antibody (anti-PV), and neuronal cell bodies were labeled using NeuroTrace 640/660. Slides were rinsed in PBS for 5 minutes and transferred into 0.01 M citric acid buffer heated to 95°C for 20 minutes during heat-induced epitope retrieval. The slides were removed, cooled, and then rinsed in PBS for 5 minutes. Excess PBS was removed, and the perimeter of each slide was traced using a Super PAP Pen (Daido Sangyo) to create a barrier. Slides were then rinsed in 0.01% PBS-Tween (Sigma–Aldrich) for 2 minutes. Blocking solution (10% donkey serum + 10% Carbo-Free blocking solution (10×) + PBS-Triton X-100 (0.3%)) was applied to each slide before incubation at room temperature for an hour. The blocking solution was suctioned off, and primary antibody was applied in the next blocking solution (1:600 anti-PV, 1:500 biotinylated WFA). A second incubation occurred overnight at 4°C. On the second day of staining, secondary antibodies were diluted in blocking solution (1:250 Alexa Fluor 488 (conjugated to streptidavin), 1:250 Alexa Fluor 594, and 1:150 NeuroTrace 640/660) and kept in the dark. The slides were rinsed in triplicate (2 minutes each) in 0.01% PBS-Tween, and then secondary antibodies applied. Slides were incubated at room temperature for 1 hour in the dark and then washed in triplicate. Excess liquid was suctioned off and coverslips (Fisher Scientific, 12-545M) were adhered using Immumount (Shandon). Slides were stored at 4°C for up to 1-week post-staining. Fluorescent imaging using GFP, Texas Red, and CY5 channels was conducted using an EVOS FLAuto2 inverted epifluorescent microscope (Invitrogen, ThermoFisher Scientific) at 10× magnification. Images were taken at a frequency of 1:48 sections and, on average, 6 images covered the lesioned tissue for every animal (~6000 μm).

### Lesion Volume and Cell Quantification

**Figure 2.**
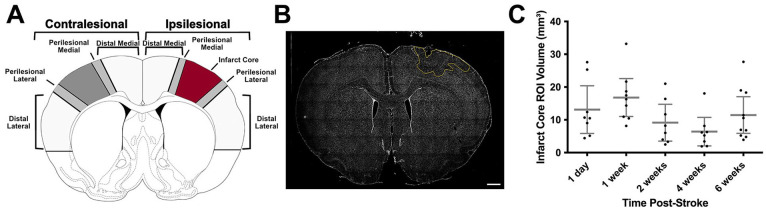
Infarct traces and ROI infarct core volumes. (A) Schematic delineating ROI. (B) Whole brain slice with ischemic core outlined in yellow. (C) Infarct core ROI volumes (intact tissue in undamaged hemisphere minus intact tissue in damaged hemisphere multiplied by section thickness and interval) across timepoints post-stroke (n = 43). Data are mean ± 95% confidence interval. Scale bar: *B* = 1000 μm.

Infarct volume was calculated by subtracting the area of the contralesional hemisphere from the intact tissue in the ipsilesional hemisphere multiplied by the thickness of each section and the interval at which sections were taken. To quantify PV+ interneurons, PNNs, and neuronal cell bodies, a novel cell-counting macro was created using Fiji/ImageJ2 software (see Supplemental Methods: Cell Counting Macro). Five different region(s) of interest (ROI) were segmented in both the ipsilesional and contralesional hemispheres (Figure 2A). The selection of these ROIs was based on studies that identified perilesional and more distal cortical regions contributing to behavioral recovery^25,30^ as well as previous PNN and PV+ quantification studies.^17,21,26^ Accordingly, ipsilesional ROIs consisting of a distal medial cortical region corresponding to the cingulate cortex; a 1000 µm wide perilesional area on the medial aspect of the infarct, the ischemic core which was primarily centered in the caudal and rostral forelimb areas as well as hindlimb motor cortex (Figure 2B); a 1000 µm perilesional area on the lateral border of the infarct and a distal lateral cortical region corresponding to the forelimb somatosensory cortex.^40^ The infarct cores were not quantified for cell density metrics, but the average infarct volume was used to parcellate the homologous regions in the unlesioned, contralesional hemisphere (Figure 2C). The average ROI areas across animals were as follows (in μm^2^): distal lateral contralesional = 78.2 ± 17.3 (mean ± standard deviation); perilesional lateral contralesional = 16.8 ± 4.68; perilesional medial contralesional = 16.5 ± 4.54; distal medial contralesional = 33.3 ± 8.65; distal medial ipsilesional = 34.9 ± 9.09; perilesional medial ipsilesional = 17.1 ± 4.30; perilesional lateral ipsilesional = 17.2 ± 5.07; distal lateral ipsilesional = 83.6 ± 19.8. We utilized the open-source deep neural net pixel classification software Ilastik (version 1.3.0) to threshold fluorescent images for quantification with ImageJ’s “analyze particles” function.^41^ Based on training of the software, Ilastik produced a binarized map of each fluorescent channel. To find the optimal training paradigm for the neural net, the perilesional lateral areas in the ipsilesional and contralesional cortex were manually counted in a subset of the animals (n = 13). Once the neural network training generated statistically similar counts between ImageJ/Fiji and manual quantification (see Supplemental Methods: Section 2, Supplemental Figure 1, Supplemental Table 2, and Supplemental Table 3), binarized segmentation maps were produced for all ROI and all included animals (N = 87). The segmentation maps of the images produced by Ilastik were quantified using Fiji/ImageJ2. Total PV+ cells, PNNs, and cell co-expression were quantified in each ROI.

### Perilesional and Distal Lesional ROI Amalgamation

We originally analyzed cell density ROI data using a repeated measures analysis of variance (ANOVA; see Supplemental Methods: Section 3). In the within-subject effects, we did not see significant interactions of perilesional/distal lesional × lateral/medial × contralesional/ipsilesional × surgery type × timepoint but we did see significant interactions of lateral/medial × contralesional/ipsilesional × surgery type × timepoint (see Supplemental Table 4). While the repeated measures ANOVA is no longer the main statistical analysis, we used the within-subject effects to inform the factors for our linear mixed models, and consequently combined the perilesional and distal lesional ROI for the rest of the analysis. For cell densities of peri and distal lesional areas, please see Supplemental Table 5.

To combine perilesional and distal lesional ROI, we summed perilesional and distal lesional cell counts and ROI areas for a given cell type (PV+, PNN, and co-label) in each region (lateral contralesional, medial contralesional, lateral ipsilesional, or medial ipsilesional) for each imaged brain section from each animal. We calculated individual imaged section densities for each ROI from these summed values. This collapsed the perilesional and distal lesional areas together, and left us with 4 ROI: lateral contralesional, medial contralesional, lateral ipsilesional, and medial ipsilesional.

For the remaining methods and analyses, the lateral cortex includes all tissue lateral to the infarct core extending to the rhinal fissure (or homologous area on the contralesional side; perilesional lateral + distal lateral), and the medial cortex means all tissue medial to the infarct core extending to the midline (or homologous area on the contralesional side; perilesional medial + distal medial).

### Cell Density Normalization

To reduce variability, we normalized ipsilesional hemisphere ROI densities to contralateral hemisphere ROI densities. Original cell density analysis using non-normalized density values can be found in Supplemental Figures 2 and 3. To normalize, we divided a given imaged section’s ipsilesional density by the corresponding contralesional density. We removed outliers that fell above the 99th percentile. For all data in each ROI (lateral contralesional, medial contralesional, lateral ipsilesional, and medial ipsilesional), we averaged densities of all imaged sections for each animal.

### Statistics

All statistical analyses were conducted using IBM SPSS Statistics for Mac (version 29). Manually traced infarct core ROI volumes were assessed using a linear model with a fixed effect of timepoint. Behavioral tasks were analyzed with a linear mixed model where the fixed effects were timepoint and surgery type and the random effect was animal ID. There was a within-subjects repeated measure of time. Normalized cell density data was also analyzed using linear mixed models, where the fixed effects were timepoint, surgery type, and medial/lateral areas (as a categorical variable). Adjustment for multiple comparisons was done using Sidak correction. Significance was set at *P* < .05 for all analyses. All values reported are estimated marginal means ±95% confidence interval.

## Results

### Infarct Volume

There were no significant differences in infarct volume across stroke groups at each timepoint, assessed using a linear model (Figure 2C).

### Behavior

**Figure 3.**
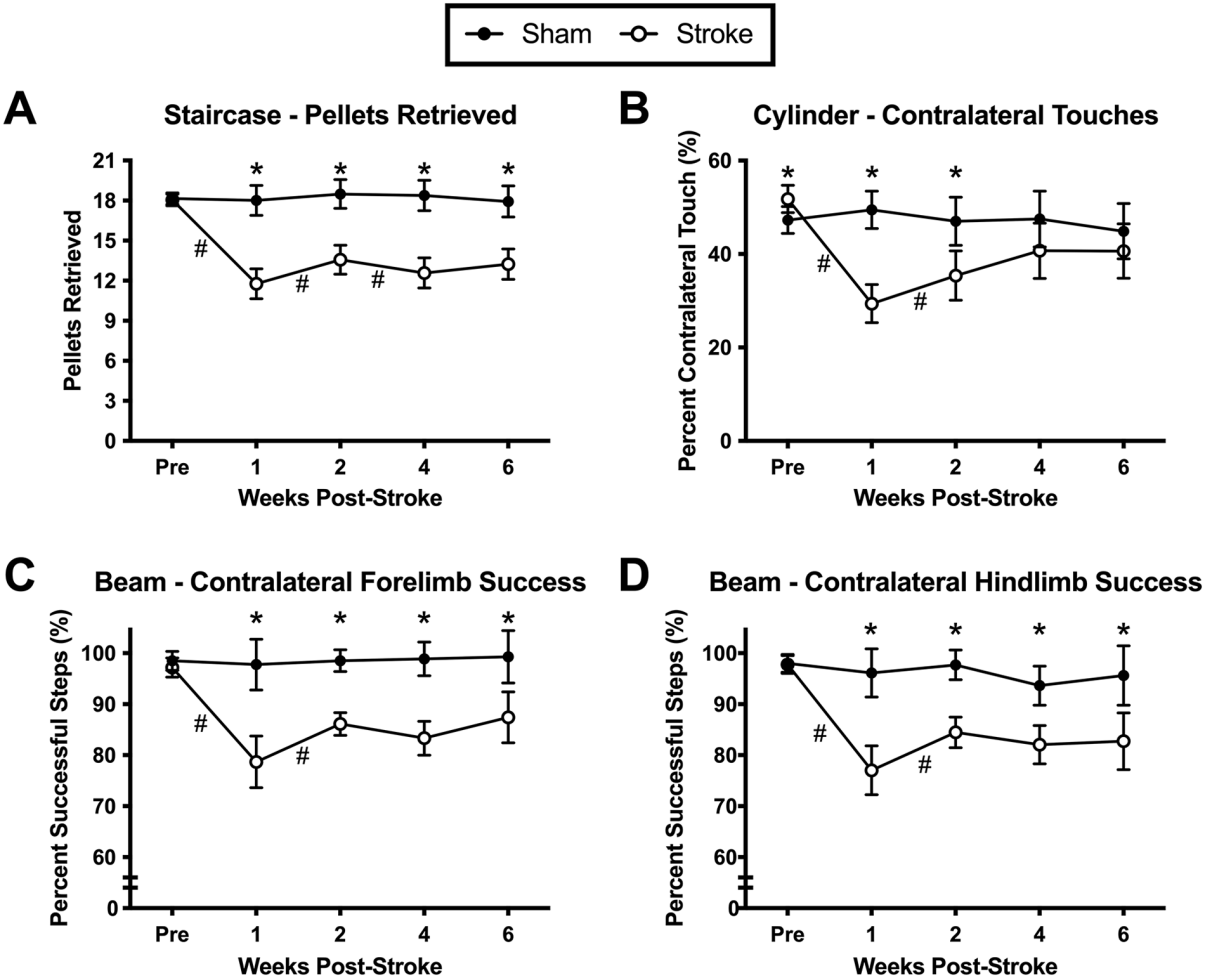
Behavioral tasks in stroke and sham groups across pre- and post-stroke timepoints. (A) Staircase task. (B) Cylinder task. (C) Beam task measured for the contralateral forelimb. (D) Beam task measured for the contralateral hindlimb. Data are mean ± 95% confidence interval. *P < .05 between groups, ^#^*P* < .05 within stroke group, comparing adjacent timepoints to each other.

In both the staircase and beam tasks, there was a significant decrease in stroke animal performance at 1, 2, 4, and 6 weeks post-stroke compared to shams (Figure 3A, C, and D; Table 1). In the cylinder task, there was a small but significant difference in performance between sham and stroke animals (% contralateral touch: sham = 47.3 ± 7.61% (mean ± standard deviation); stroke = 51.8 ± 8.16%) at the pre-stroke timepoint as well as significantly worse performance in stroke animals compared to sham animals at 1 and 2 weeks post-stroke (Figure 3B). Within the stroke group, all behavioral tasks showed a significant reduction in performance between pre-stroke and 1-week post-stroke, and a significant increase in performance between 1 and 2 weeks post-stroke. Staircase testing revealed a significant decrease in performance between 2 and 4 weeks post-stroke within the stroke animal cohort.

**Table 1.** Statistical Data for All Behavioral Tasks.

Behavioral task	*F*	df	*P*
Staircase	20.273	4, 36.262	<.001
Cylinder	13.656	4, 37.126	<.001
Beam (forelimb)	11.692	4, 27.917	<.001
Beam (hindlimb)	12.171	4, 30.576	<.001

### PV+ Interneuron Density

**Figure 4.**
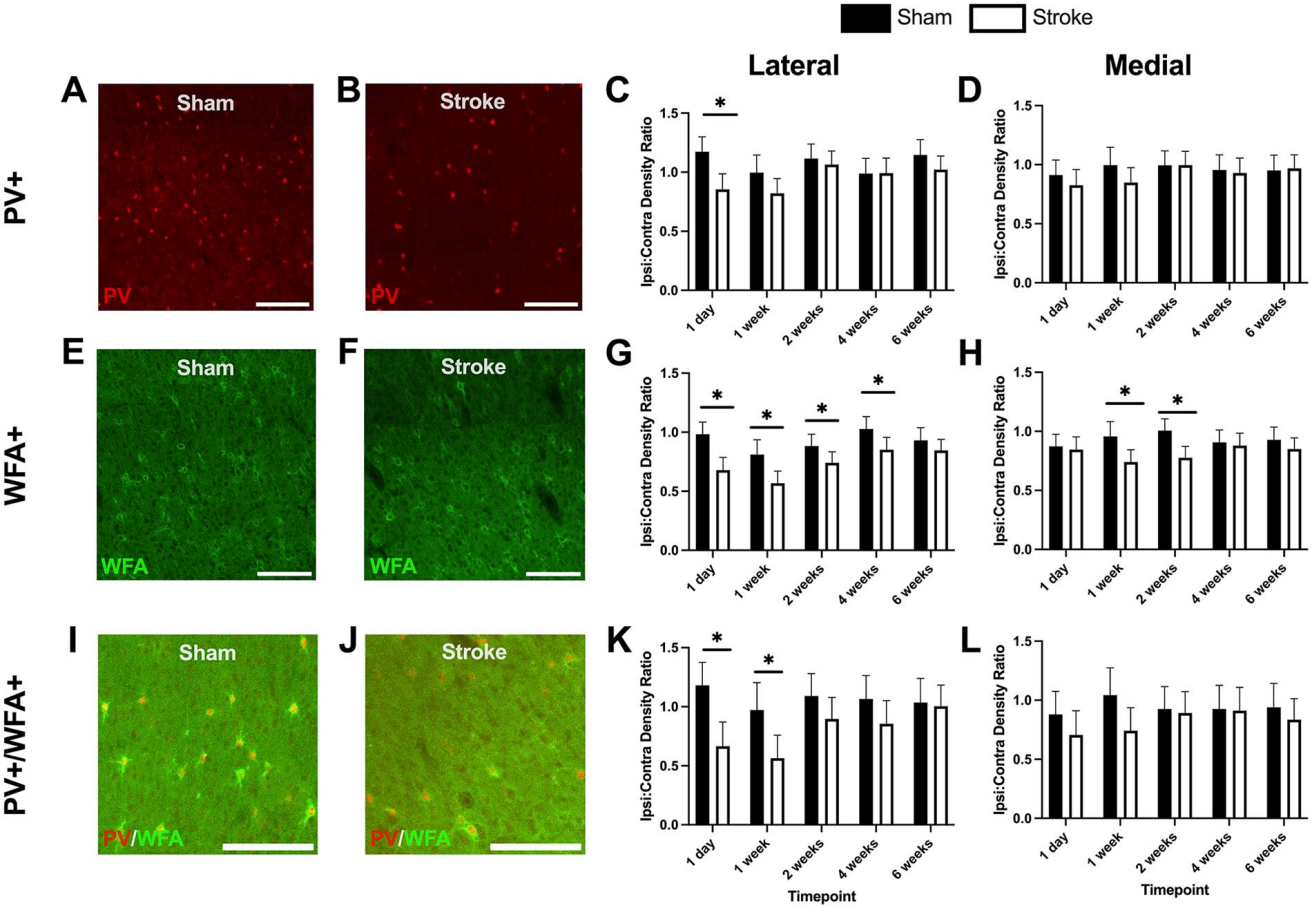
PV+ interneuron, WFA+, and WFA+/PV+ co-label ratios (ipsilesional cell density:contralesional cell density) in different ROI across timepoints in stroke and sham cohorts. (A) Representative image of PV+ interneurons in a sham animal at the 1 week timepoint. (B) Representative image of PV+ interneurons in a stroke animal at the 1 week timepoint. (C) Lateral PV+ interneuron normalized density ratios. (D) Medial PV+ interneuron normalized density ratios. (E) Representative image of WFA+ cells in a sham animal at the 1 week timepoint. (F) Representative image of WFA+ cells in a stroke animal at the 1 week timepoint. (G) Lateral WFA+ cell normalized density ratios. (H) Medial WFA+ cell normalized density ratios. (I) Representative image of co-labeled PV+ and WFA+ cells in a sham animal at the 1 week timepoint. (J) Representative image of co-labeled PV+ and WFA+ cells in a stroke animal at the 1 week timepoint. (K) Lateral co-labeled cell normalized density ratios. (L) Medial co-labeled cell normalized density ratios. Data are mean ± 95% confidence interval. Scale bars: A, B, E, F, I, and J, 200 μm. *P < .05, comparing sham and stroke cohorts.

Representative images of sham and stroke PV+ interneurons in the ipsilesional cortex at the 1-week timepoint are shown in Figure 4A and B, respectively. There was a significant decrease in PV+ interneuron density at 1-day post-stroke in the lateral ipsilesional area compared to sham animals. No significant differences were seen at subsequent timepoints. In the medial ipsilesional area, there were no significant differences seen between sham and stroke groups at any timepoints. In our original density analysis (see Supplemental Results: Section 1 and Supplemental Figure 2), we observed a reduction of PV+ interneurons in the medial ipsilesional area at 1 week post stroke, which did not appear when we normalized the data to the contralesional hemisphere.

### PNN Density

Representative images of PNNs from the ipsilesional cortex of sham and stroke animals at the 1-week timepoint are shown in Figure 4E and F. In the lateral ipsilesional area, there was a significant reduction in PNN density at 1 day, 1 week, 2 weeks, and 4 weeks post-stroke. By 6 weeks, sham and stroke PNN densities were comparable. In the medial ipsilesional area, there was a significant reduction at 1-week post-stroke and 2 weeks post-stroke. In the non-normalized density analysis (see Supplemental Results: Section 2 and Supplemental Figure 2), there were no significant differences at any timepoints, demonstrating that reducing variability via normalization to the contralesional hemisphere revealed some additional effects.

### PV+/PNN Co-Label Cell Density

Examining the densities of PV+ interneurons and PNNs independently is important to identify fluctuations in each cell type over time post-stroke. However, it is also important to look at co-expression of PV+ interneurons and PNNs since PNNs may modulate PV+ interneuron inhibitory signaling^19,28^ but see Faini et al.^42^

Representative images illustrating sham and stroke PV+/PNN co-labeling in the ipsilesional cortex at the 1-week timepoint are shown in Figure 4I and J. There was a significant decrease at 1 day and 1-week post-stroke in lateral ipsilesional PV+/PNN co-labeled cell density. There were no significant differences between sham and stroke groups over time in the medial ipsilesional area. The non-normalized co-labeled cell density data showed an effect at 4 weeks in the lateral ipsilesional area and at 1 week in the medial ipsilesional area (see Supplemental Results: Section 3 and Supplemental Figure 3).

## Discussion

We examined the spatial-temporal distribution of PV+ interneurons and PNNs during the critical window of post-stroke recovery using a novel counting paradigm that allowed for widespread quantification of PV+ interneuron and PNN densities across the cortex of both hemispheres. The value of this automated quantification approach is underscored by findings showing the substrates of recovery involve not only cortical regions distal to perilesional cortex^30^ but subcortical, brainstem and spinal cord circuits as well.^43-46^ Future efforts to identify the biological substrates of recovery will require a much broader sampling of brain regions than the peri-infarct cortex. This process will be facilitated by an automated counting method akin to that used in the present study.

### PV+ Interneuron Density Is Decreased in the Lateral Ipsilesional Cortex at 1 Day Post-Stroke

We found that PV+ interneuron density decreased significantly in the lateral ipsilesional cortex 1-day post-stroke, with comparable densities to sham animals at subsequent timepoints. The medial ipsilesional cortex was unaffected. With 1 exception,^47^ most studies have not reported reductions in contralesional^10,29^ PV+ expression. The reduction of PV+ interneurons as early as 1-day post-stroke demonstrates that PV+ are reduced immediately after stroke in the lateral ipsilesional area. Some studies using a transient ischemic stroke model have found reduced PV+ expression in ipsilesional areas at 24 hours post-stroke.^48^ Others have seen a more delayed^29^ or prolonged^47^ decrease in PV+ expression. Regardless, the reduction in PV+ interneurons 1 day after stroke in the lateral ipsilesional area suggests that there is an early, temporally-dependent modulation of GABA-mediated inhibition. Decreases in GABA signaling and PV+ interneurons have been linked to higher levels of neuroplasticity and increases in long term potentiation, which in turn can affect behavioral recovery.^22,29,49^ Although we do not see a strong link between PV+ interneuron reduction and behavioral recovery in our study, it may be that the most important factor to consider is the interplay between PNNs and PV+ cells reflected in the expression of co-labeled PV+ and PNN cells over time after stroke.

### PNN Density is Decreased in the Ipsilesional Hemisphere Following Stroke

PNNs are implicated in critical periods of neurodevelopment and other forms of plasticity^9,11,14-16,50^ including post-stroke recovery.^10^ While PV+ interneuron density underwent a more transient reduction post-stroke, PNNs showed consistent reductions in density up to 4 weeks post-stroke in the lateral ipsilesional region (Figure 4G) and reduction at 1 and 2 weeks post-stroke in the medial ipsilesional region (Figure 4H).

Other studies have also reported similar results at different timepoints during the critical post-stroke window of neuroplasticity.^7,10,26,29^ In our study, the stroke-induced reduction in PNNs lasted between 4 and 6 weeks post-stroke, which is in agreement with other findings.^26,29^ Although Karetko-Sysa et al^26^ found a significant decrease in PNN density in contralateral homotopic areas to the stroke, this was not evident in our experiments or others in the literature.^10,29^ Such inconsistences may be due to the use of different stroke models, or variations in infarct size and location.

Reduced PNN density creates a permissive environment for neuroplasticity.^10,19^ We observed a consistent reduction in PNNs in the lateral ipsilesional area between 4 and 6 weeks post-stroke. Other animal studies have shown that the greatest stroke recovery occurs in the first month after stroke,^7,51^ which complements the time course of PNN reduction reported herein. However, our behavioral results did not precisely follow the course of PNN reduction. We instead saw a plateau in behavior 2 weeks post-stroke. This suggests that, while PNN degradation creates a permissible environment for neuroplasticity, another factor is influencing behavioral recovery in our study.

### Behavioral Recovery Corresponds to a Reduced Density of Co-Labeled Cells

The concurrent decline of PV+ interneurons and PNNs potentially creates a state of heightened neuroplasticity post-stroke, which may allow for enhanced spontaneous functional recovery. Consistent with this interpretation is the observation that PNNs were chronically decreased and the greatest reductions in PV+ expression as well as PV+/PNN co-labeling occurred primarily within the first 1 to 2 weeks post-stroke. This reduction coincides with the time frame of spontaneous behavioral recovery as indicated by the rapid slope changes in recovery evident at 1-week post-stroke. Thus, the concurrent reduction in PV+ interneurons and PNNs together with reduced numbers of PV+ cells enveloped by PNNs may provide an optimal condition for post-stroke recovery possibly resetting the cortical excitatory-inhibitory balance in favor of excitation.^28^ This scenario opens possibilities for extending or re-opening the critical window of post-stroke recovery. There is some evidence suggesting that such an approach is feasible. Several studies have administered a GABA_A_ receptor inverse agonist and found that this increased behavioral recovery.^22,24,29^ Another group found that downregulating tonic GABA signaling and upregulating phasic GABA signaling improved stroke recovery outcomes in a mouse model.^23^ Overall, the evidence for inhibiting GABA signaling and components of the extracellular matrix such as PNNs to improve and extend the window for behavioral recovery after stroke looks promising.^22-24,28,52^

## Limitation

Our experiments utilized young, adult male rats and thus it remains to be determined if the observed results would generalize to female animals or older animals with disease co-morbidities. Additionally, it is unclear if the improved behavioral performance observed in the stroke group is due to pure recovery (ie, restitution of function), compensation, or some combination of the two. Future work should utilize kinematic analyses of the behavioral tasks to distinguish between these possibilities.^43,53^

## Conclusions

Overall, our results suggest that spontaneous behavioral recovery occurs in parallel with a reduction of PV+/PNN co-labeled cells. Reductions in PV+ interneurons and PNNs are implicated in numerous forms of plasticity including developmental critical periods, and the changes in co-labeled PV+/PNN markers coincided with the beginning of spontaneous behavioral recovery observed 1-2 weeks post-stroke. An early but transient reduction in PV+ expression in the lateral ipsilesional cortex and reductions in PNNs up to 4 weeks in the lateral ipsilesional cortex and at 1 and 2 weeks post-stroke in the medial ipsilesional cortex may create a permissive state for neuroplasticity and, consequently, behavioral recovery. Our data provide additional evidence linking PV+ interneurons and associated PNNs with the critical window of post-stroke recovery. In the event that reopening and/or extending the critical window in humans^54^ can be achieved by decreasing inhibitory signaling, it may be possible to achieve more rapid and complete restoration of post-stroke function.

## Supplemental Material

sj-docx-1-nnr-10.1177_15459683241309567 – Supplemental material for Post-Stroke Recovery in Relation to Parvalbumin-Positive Interneurons and Perineuronal Nets Supplemental material, sj-docx-1-nnr-10.1177_15459683241309567 for Post-Stroke Recovery in Relation to Parvalbumin-Positive Interneurons and Perineuronal Nets by Lydia M. Kuhl, Matthew S. Jeffers, Nicolay Hristozov, Sudhir Karthikeyan, Matthew W. McDonald, Aisha Hufnagel, Anthony Carter, Numa Dancause and Dale Corbett in Neurorehabilitation and Neural Repair
