# Supplementary material for: Post-Stroke Recovery in Relation to Parvalbumin-Positive Interneurons and Perineuronal Nets
Source: Neurorehabil Neural Repair. 2025 Jan 16;39(4):286–96. doi: 10.1177/15459683241309567 (PMC11982584; doi:10.1177/15459683241309567)
Supplement: sj-docx-1-nnr-10.1177_15459683241309567 – Supplemental material for Post-Stroke Recovery in Relation to Parvalbumin-Positive Interneurons and Perineuronal Nets [file sj-docx-1-nnr-10.1177_15459683241309567.docx]

Supplemental Methods: Section 1: Group Size

Preliminary power analysis indicated an effect size for reduction of PNNs in the perilesional area of d=2.25. This large effect size would require n=6 animals per group to achieve 95% power to detect the effect in our study. Based on preliminary data, we elected to take a conservative approach in case this effect size was an overestimate and included a minimum of n=8 animals per group in the present study.

Supplemental Methods: Table 1. Triple-label immunofluorescence staining materials.

| Blocking Solution | Company | Product ID |
| --- | --- | --- |
| Normal Donkey Serum | Jackson ImmunoResearch | 017-000-121 |
| Carbo-Free Blocking Solution | Vector Laboratories | SP5040 |
| Triton-X100 | Sigma-Aldrich | X100 |
| Anti-Parvalbumin | Sigma-Aldrich | SAB4200545 |
| Wisteria Floribunda Lectin | Vector Laboratories | B-1355 |
| Alexa Fluor® 594 | Jackson ImmunoResearch | 715-585-150 |
| Alexa Fluor® 488 | Jackson ImmunoResearch | 016-540-084 |
| NeuroTrace™ 640/660 | Invitrogen | N21483 |

Supplemental Methods: Section 2: Manual Counting of PNNs and PV+ cells and Ilastik Training

Manual counts were performed on 13 animals using the 1000μm^2^ perilesional lateral contralesional area and the perilesional lateral ipsilesional area (Supplemental Figure 1A). Comparing the manual counts of PNNs to different numbers of training samples, it was found that after training our pixel classification neural net using 40 images, the number of cells counted using Ilastik was not significantly different from the manual count (Supplemental Figure 1B). However, for PV+ interneurons this level of accuracy was not attained until after 50 images had been used for training the neural net (Supplemental Figure 1C). This analysis demonstrates the quality of our automated quantification paradigm, enabling quantification of PV+ interneurons and PNNs across the entire cortex, which has not been previously performed post-stroke, due to the time-consuming nature of manual counting.


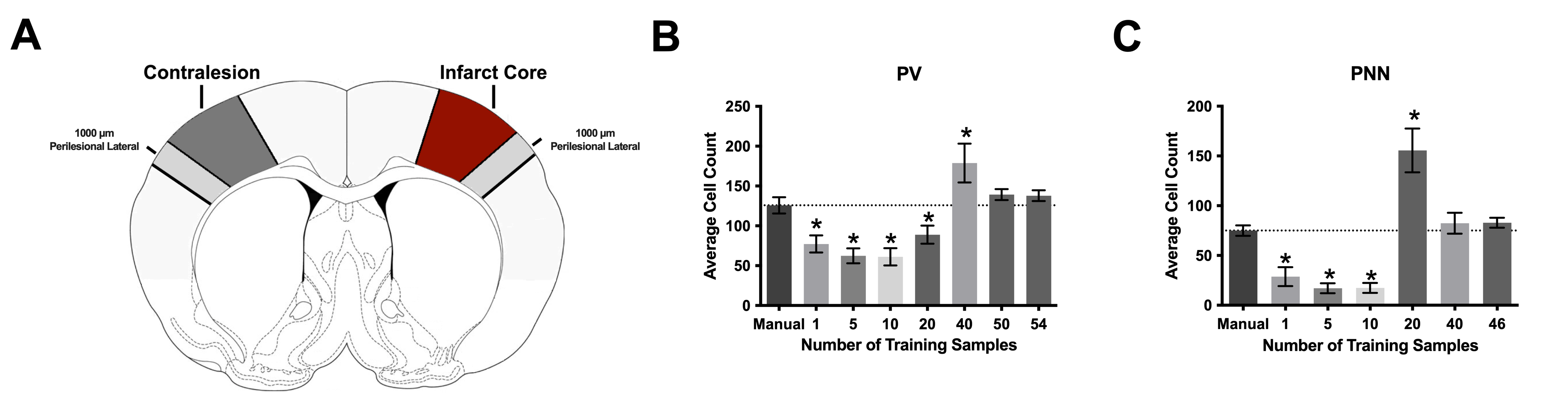


**Supplemental Figure 1. Manual counting compared to increasing amounts of Ilastik training.** (A) Schematic delineating the areas that were manually counted and used for assessing quality of Ilastik training. (B) Average manual counts of PV+ interneurons compared to average cell counts for increasing numbers of images used in neural net training. Lateral contra and ipsilesional test band cell counts were collapsed together, and cell counts were averaged. Dashed line indicates the mean of the manual counts. (C) Average manual counts of PNNs compared to average cell counts for increasing numbers of images used in neural net training. Lateral contra and ipsilesional test band cell counts were collapsed together, and cell counts were averaged. Dashed line indicates the mean of the manual counts. Data are mean ± 95% CI. * p < 0.05 comparing manual counting to all other training paradigms.

Supplemental Table 2. PV+ cell training samples for Ilastik neural network. All table cells display the raw summed cell count across all animals in the training sample.

|  | **PV Hand Count (# cells)** | PV Neural Net 1 Sample (# cells) | PV Neural Net 5 Samples (# cells) | PV Neural Net 10 Samples (# cells) | PV Neural Net 20 Samples (# cells) | PV Neural Net 40 Samples (# cells) | PV Neural Net 50 Samples (# cells) | PV Neural Net 54 Samples (# cells) |
| --- | --- | --- | --- | --- | --- | --- | --- | --- |
| **Perilesional lateral contralesional** | **11035** | 6899 | 5661 | 5468 | 7995 | 15964 | 12531 | 12496 |
| **Perilesional lateral ipsilesional** | **9840** | 5925 | 4700 | 4683 | 6733 | 13726 | 10588 | 10391 |

Supplemental Table 3. PNN+ cell training samples for Ilastik neural network. All table cells display the raw summed cell count across all animals in the training sample.

|  | **PNN Hand Count (# cells)** | PNN Neural Net 1 Sample (# cells) | PNN Neural Net 5 Samples (# cells) | PNN Neural Net 10 Samples (# cells) | PNN Neural Net 20 Samples (# cells) | PNN Neural Net 40 Samples (# cells) | PNN Neural Net 46 Samples (# cells) |
| --- | --- | --- | --- | --- | --- | --- | --- |
| **Perilesional lateral contralesional** | **6410** | 2447 | 1557 | 1572 | 13198 | 8096 | 8134 |
| **Perilesional lateral ipsilesional** | **6046** | 2316 | 1280 | 1316 | 12625 | 5582 | 5634 |

Supplemental Methods: Section 3: Repeated measures ANOVA methodology

Densities of PV+ interneurons, PNNs, and the percentages of PNN and PV+ cells were assessed using repeated-measures ANOVAs. The Greenhouse-Geisser correction was applied in cases where the assumption of sphericity was violated. Within-subject factors were: contralesional vs. ipsilesional, perilesional (<1000μm from infarct) vs. distal (>1000μm from infarct), and lateral vs. medial, and between-subject factors were surgery type and timepoint. The interactions of interest for post-hoc analyses were contralesional/ipsilesional vs. lateral/medial vs. surgery type vs. time. Post-hoc analysis using Sidak-corrected multiple t-tests revealed significant interactions between these variables. Significance was set at p<0.05 for all analyses and was also the correction level for all post-hoc analyses. All values reported are estimated marginal means $\pm$ 95% confidence interval.

Supplemental Table 4. Omnibus statistics for perilesional/distal lesional*lateral/medial*contralesional/ipsilesional*surgery type*timepoint interactions for the repeated measures ANOVA.

| Variable | F | p |
| --- | --- | --- |
| PV+ density | 1.832 | 0.131 |
| WFA+ density | 0.710 | 0.588 |
| PV+/WFA+ co-label density | 0.985 | 0.421 |

Supplemental Table 5. Peri and distal lesional cell densities for PV+, PNNs, and co-labelled PV+/PNN cells.

| **Region** | **Perilesional (mean ± std)** | **Distal lesional (mean ± std)** |
| --- | --- | --- |
| PV+ Lateral Contra | 53.36 ± 14.34 | 47.56 ± 14.43 |
| PV+ Lateral Ipsi | 46.44 ± 16.61 | 44.72 ± 15.04 |
| PV+ Medial Contra | 52.32 ± 13.78 | 44.08 ± 14.79 |
| PV+ Medial Ipsi | 46.16 ± 14.70 | 42.70 ± 15.65 |
| PNN Lateral Contra | 32.43 ± 11.69 | 29.06 ± 10.44 |
| PNN Lateral Ipsi | 25.72 ± 12.77 | 24.88 ± 10.45 |
| PNN Medial Contra | 29.23 ± 11.64 | 23.93 ± 9.738 |
| PNN Medial Ipsi | 24.46 ± 11.20 | 22.74 ± 10.01 |
| PV+/PNN Lateral Contra | 13.67 ± 5.045 | 11.63 ± 4.594 |
| PV+/PNN Medial Contra | 13.18 ± 5.114 | 9.650 ± 4.130 |
| PV+/PNN Lateral Ipsi | 10.17 ± 5.57 | 10.11 ± 4.391 |
| PV+/PNN Medial Ipsi | 10.05 ± 4.805 | 8.945 ± 4.038 |


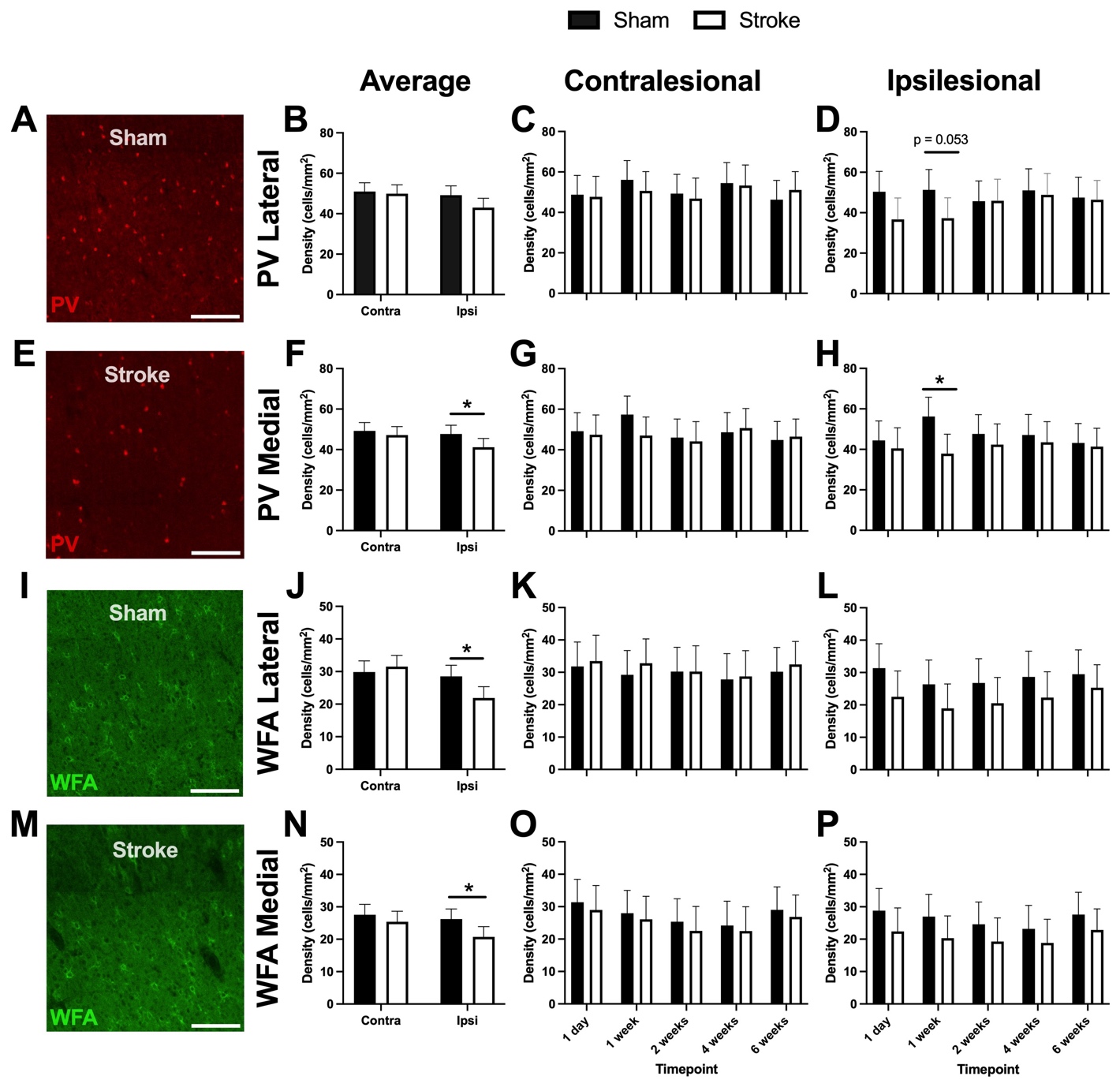


**Supplemental Figure 2.** **PV+ interneuron and WFA+ cell densities (cells/mm^2^) in different ROI across timepoints in stroke and sham cohorts.** (A) Representative image of PV+ interneurons in a sham animal at the 1 week timepoint. (B) Lateral contra- and ipsilesional PV+ interneuron densities with data collapsed across time and averaged. (C) Lateral contralesional PV+ interneuron densities. (D) Lateral ipsilesional PV+ interneuron densities. (E) Representative image of PV+ interneurons in a stroke animal at the 1 week timepoint. (F) Medial contra- and ipsilesional PV+ interneuron densities with data collapsed across time and averaged. (G) Medial contralesional PV+ interneuron densities. (H) Medial ipsilesional PV+ interneuron densities. (I) Representative image of WFA+ cells in a sham animal at the 1 week timepoint. (J) Lateral contra- and ipsilesional WFA+ cell densities with data collapsed across time and averaged. (K) Lateral contralesional WFA+ cell densities. (L) Lateral ipsilesional WFA+ cell densities. (M) Representative image of WFA+ cells in a stroke animal at the 1 week timepoint. (N) Medial contra- and ipsilesional WFA+ cell densities with data collapsed across time and averaged. (O) Medial contralesional WFA+ cell densities. (P) Medial ipsilesional WFA+ cell densities. Data are mean ± 95% CI. Scale bars: D, H, L, P, 200μm. * p < 0.05, comparing sham and stroke cohorts.


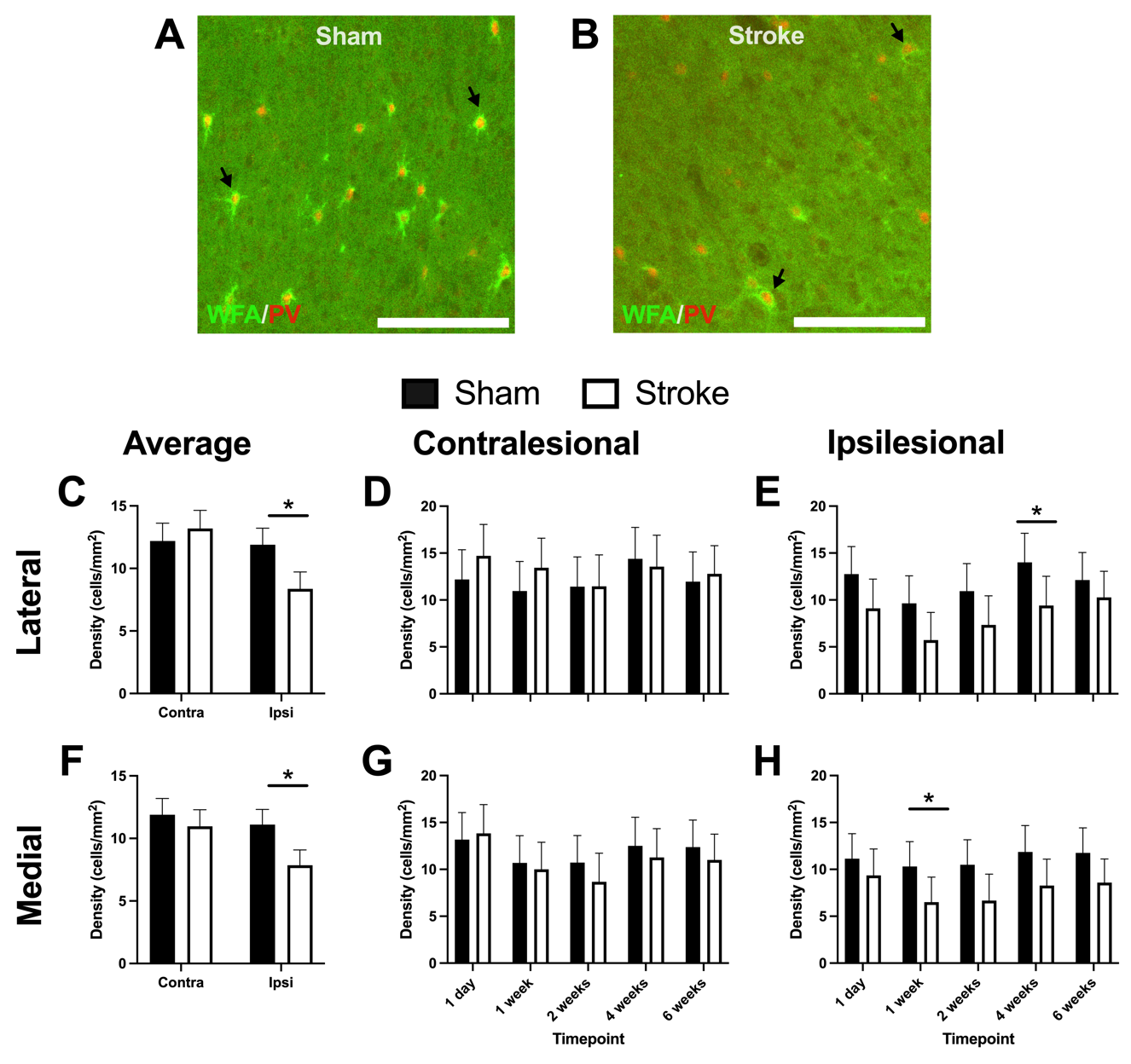


**Supplemental Figure 3. Density of WFA+ /PV+ co-labelled cells in different ROI across timepoints in stroke and sham cohorts.** (A, B) Representative images of co-labelled cells in sham and stroke animals at the 1 week timepoint. Arrowheads indicate examples of co-labelled cells. (C) Lateral contra- and ipsilesional densities of co-labelled cells with data collapsed across time and averaged. (D) Lateral contralesional densities of co-labelled cells. (E) Lateral ipsilesional densities of co-labelled cells. (F) Medial contra- and ipsilesional densities of co-labelled cells with data collapsed across time and averaged. (G) Medial contralesional densities of co-labelled cells. (H) Medial ipsilesional densities of co-labelled cells. Data are mean ± 95% CI. Scale bars: A, B, 200μm. * p < 0.05, comparing sham and stroke cohorts.

Supplemental Results: Section 1: PV+ Interneuron Density

The omnibus analysis revealed that densities in the perilesional and distal areas were not significantly different, so these areas were combined for all following cell density analyses (see Supplemental Table 2). For the remaining analyses, the lateral cortex includes all tissue lateral to the infarct core extending to the rhinal fissure (or homologous area on the contralesional side; perilesional lateral + distal lateral), and the medial cortex means all tissue medial to the infarct core extending to the midline (or homologous area on the contralesional side; perilesional medial + distal medial). Representative images of sham and stroke PV+ interneurons in the ipsilesional cortex at the 1 week timepoint are shown in Supplemental Figure 2A and 2E, respectively. We collapsed lateral and medial PV+ interneuron densities in the contralesional and ipsilesional cortex across all timepoints and took the average (Supplemental Figure 2B, F). There was a significant decrease in PV+ interneuron density in stroke animals in the medial ipsilesional cortex. This difference was not seen in the lateral ipsilesional cortex or in the lateral and medial contralesional cortex when collapsed across timepoints. At all timepoints, there were no significant differences between stroke and sham animals in the lateral and medial contralesional cortex (Supplemental Figure 2C, G). There was a reduction, albeit non-significant in PV+ interneuron density in the lateral ipsilesional cortex at 1 week post-stroke (p = 0.053, Supplemental Figure 2D). There was a significant decrease in PV+ interneuron density in the medial ipsilesional cortex at 1 week post-stroke (Supplemental Figure 2H).

Supplemental Results: Section 2: PNN Density

Representative images of PNNs from the ipsilesional cortex of sham and stroke animals at the 1 week timepoint are shown in Supplemental Figure 2I, M. PNN density was collapsed across all timepoints and averaged in the contralesional and ipsilesional cortex. A significant decrease in PNN density was observed in stroke animals in the lateral and medial ipsilesional cortex (Supplemental Figure 2J, N). This was not seen in the lateral or medial contralesional cortex; additionally, there were no significant differences in PNN density between sham and stroke groups at any timepoint in either the lateral or medial contralesional cortex (Supplemental Figure 2K, O). There were no significant interactions between time and surgery type within the ipsilesional cortex (Supplemental Figure 2L, P). Overall, PNN density was reduced in the medial and lateral ipsilesional cortex post-stroke, indicating a widespread chronic effect in the ipsilesional hemisphere.

Supplemental Results: Section 3: PV+/PNN co-labelled cell density

Representative images illustrate sham and stroke PNN and PV co-labelling in the ipsilesional cortex at the 1 week timepoint (Supplemental Figure 3A, B). When co-label density was collapsed across timepoints, the stroke cohort in both the lateral and medial ipsilesional cortex had a significantly lower density of PNN/PV+ co-labelled cells compared to the sham cohort (Supplemental Figure 3C, F). There were no significant differences between stroke and sham groups at any timepoint in the medial and lateral contralesional cortex (Supplemental Figure 3D, G). In the lateral ipsilesional cortex, there was a significant reduction of co-labelled cell density in stroke animals at 4 weeks post-stroke (Supplemental Figure 3E). In the medial ipsilesional cortex, there was a significant reduction of co-labelled cell density in stroke animals at 1 week post-stroke (Supplemental Figure 3H).
